# CHOmine: an integrated data warehouse for CHO systems biology and modeling

**DOI:** 10.1093/database/bax034

**Published:** 2017-04-22

**Authors:** Matthias P. Gerstl, Michael Hanscho, David E. Ruckerbauer, Jürgen Zanghellini, Nicole Borth

**Affiliations:** 1 Department of Biotechnology, University of Natural Resources and Life Sciences, Muthgasse 19, 1190 Vienna, Austria; 2 Austrian Centre of Industrial Biotechnology (ACIB), Muthgasse 11, 1190 Vienna, Austria

## Abstract

The last decade has seen a surge in published genome-scale information for Chinese hamster ovary (CHO) cells, which are the main production vehicles for therapeutic proteins. While a single access point is available at www.CHOgenome.org, the primary data is distributed over several databases at different institutions. Currently research is frequently hampered by a plethora of gene names and IDs that vary between published draft genomes and databases making systems biology analyses cumbersome and elaborate. Here we present CHOmine, an integrative data warehouse connecting data from various databases and links to other ones. Furthermore, we introduce CHOmodel, a web based resource that provides access to recently published CHO cell line specific metabolic reconstructions. Both resources allow to query CHO relevant data, find interconnections between different types of data and thus provides a simple, standardized entry point to the world of CHO systems biology.

**Database URL:** http://www.chogenome.org

## Introduction

Chinese hamster ovary (CHO) cells have been used for production of biotherapeutic proteins since 1985 (1), with the biopharmaceutical market of CHO derived products grown to > 100 billion US$ by 2013 (2). Due to the importance of this cell line for the biopharma industry a plethora of -omics data was generated during the last years. Today a sequenced CHO-K1 genome (3) and two Chinese hamster genomes (4, 5) are available. Unfortunately, these draft genomes are not consistent in the usage of gene IDs for annotation while other databases, such as UniProt use again other IDs. To overcome some of the difficulties that are associated with connecting such diverse and large data sets, special data warehouses like BioMart (6) or InterMine (7) were developed and already exist for important model organisms, like mouse (8) or fly (9). These solutions provide interfaces to search for information in a user friendly way and enable to connect different databases for a given gene, thus providing all the available information from a single entry point. Currently, the CHO community accesses relevant data via www.CHOgenome.org (10), which hosts all published information, however does not provide links between different data types. Therefore, we introduce CHOmine, an InterMine based data warehouse for CHO data that connects gene information to each other and provides links to outside websites. The resource also fully integrates a recently published consensus genome-scale metabolic reconstruction of different CHO cell lines (11).

## Materials and methods

**Figure 1.**
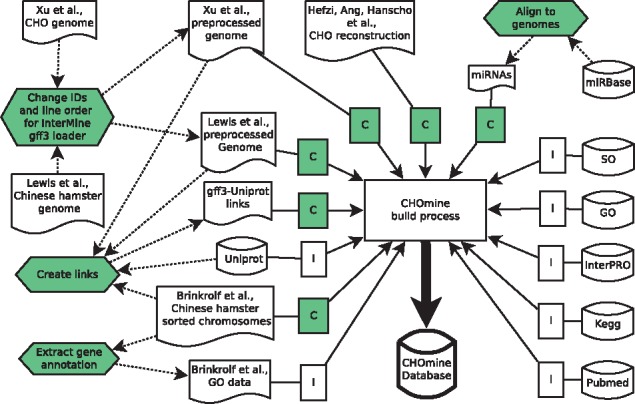
CHOmine building pipeline. Automatically downloaded files or links for every new CHOmine version. File published or created in a preprocessing step. CHOmine specific preprocessing steps. CHOmine specific data loader. InterMine data loader. Dashed arrows indicate preprocessing steps. All other arrows indicate CHOmine building steps.

CHOmine is based on the latest stable version of InterMine (7) and runs on the latest stable Debian operating system. Data is stored in a PostgreSQL database which is directly installed from the Debian repository together with the Apache Tomcat^®^ webserver. The Java Development Kit was downloaded from Oracle. InterMine provides many predefined data loader, which we used for importing data from UniProt (12), InterPro (13), KEGG (14) and PubMed (15) as well as for loading sequence ontologies (16) and gene ontologies (17). To handle the unusual situation of loading three genomes for an organism, some preprocessing steps were required as well as the creation of extended or new importer classes (Figure 1). As the GFF3 files from (3) and (5) contain the same IDs for different genes, artificial IDs were assigned to all genes. Furthermore, we created a file that links genes of the three genomes to their corresponding protein of UniProt. New created importer classes enabled us to load all three genomes as well as the upload of gene to protein links from the previously created linkage file. Gene ontology information provided by Brinkrolf *et al.* (4) was extracted from the GFF3 file and formatted, so that the InterMine GO-annotation data loader can import it. Furthermore, we downloaded miRNAs from miRBase (18) and aligned them to the three genomes using Bowtie (19), and imported the result to CHOmine by another new importer class. The pipeline for building the current version of CHOmine can be found at https://github.com/chomine/chomine.

### Genome-scale metabolic reconstructions

To connect the consensus model for the Chinese hamster and metabolic models of different CHO cell lines (11), a data loader for reading the SBML files was added to CHOmine. In order to further improve the user experience when analyzing the metabolic reconstructions, a second webpage, called CHOmodel, was created. CHOmodel makes use of the PHP framework Laravel and a separate PostgreSQL database. Materialized views were prepared in the PostgreSQL database to allow efficient browsing through the information. As the CHOmodel webpage was developed in parallel to CHOmine, we added links from CHOmodel to CHOmine and vice versa.

## Discussion and conclusion

Although the amount of data does not yet reflect the entire published dataset of CHO cells, CHOmine provides a comprehensive overview and thus a valuable resource for finding CHO relevant data. As CHOmine is based on InterMine, all of its powerful features can be used. Data can be easily searched and downloaded or different APIs can be used to access the data by scripts. CHOmine already includes different data types, like genome information, proteins, miRNAs and metabolic models, with links to many outside databases. CHOmine will be actively improved and new data types included in future versions. Raw data for older versions of CHOmine will be kept at least for two years and will be made available via the contact form of CHOmine. We are convinced that this resource will become the first point where to search for information when working with CHO cells.

## Funding

Austrian BMWFW, BMVIT, SFG, Standortagentur Tirol, Government of Lower Austria and ZIT through the Austrian FFG-COMET-K2 Funding Program.

*Conflict of interest*. None declared.
